# Dr. George S. Fouke

**Published:** 1896-06

**Authors:** 


### Obituary.

### DR. GEORGE S. FOUKE.

Dr. George S. Fouke died after an illness of several weeks, at his residence in Westminster, Md., April 9th, 1896. Dr. Fouke was born in Shepherdstown, West Virginia, on October 11, 1817, was educated in the schools there; entered Gettysburg (Pa.) College, and after graduation was appointed principal of the Waynesboro academy. While occupying this position he prepared himself for the Luthern ministry and was graduated from the Gettysburg Theological Seminary. Shortly after he was compelled to retire from the ministry, owing to throat trouble. He resumed teaching and at the same time studied dentistry. In 1848 he moved to Westminster and began the practice of his profession there, also visiting at stated times the villages in the surrounding country, as was his custom in those days. In November, 1854, he was chosen dentist to New Windsor College, St. Joseph's and Mt. St. Mary's Colleges, at Emmitsburg, retaining the last two up to a short time previous to his death. Having been in practice so long at the above institutions he had an extended acquaintance, and frequently received social letters from former students in various parts of the country.

Dr. Fouke was married twice. By his first wife he has one daughter living, Mrs. Dr. H. X. Bonbrake, of Chambersburg, Pa.; his second wife died suddenly on June 19, 1894, leaving four children—Messrs. Harvey H., George D., Frank W., and O. Prescott.

Dr. Fouke was of a retiring disposition, fond of study and full of reminiscences. For a number of years he was a visitor to the Baltimore College of Dental Surgery, a member of the State Board of Examiners of Dental Surgery, and had published many professional papers. In religion he was an Episcopalian, and had been a member of the Vestry of Ascension Church of Westminister, frequently; in politics he was a Democrat; had served several times as a member of the City Council, and was always ready to help the place of his adoption; he was also a member of Door-to-Virtue Lodge, A. F. and A. M. In his home as husband and father, he was kind and indulgent; as a friend, genial and social, and was an excellent neighbor.

